# Stability of infundibular dilatations: a single center follow-up study and systematic review of the literature

**DOI:** 10.1007/s00701-024-05890-w

**Published:** 2024-01-30

**Authors:** Jeremias Tarkiainen, Liisa Pyysalo, Tero Hinkka, Juha-Pekka Pienimäki, Antti Ronkainen, Juhana Frösen

**Affiliations:** 1 Department of Neurosurgery, Tampere University Hospital and University of Tampere, Tampere, Finland; 2 Hemorrhagic Brain Pathology Research Group, Faculty of Medical Technology and Health Sciences, Tampere University, Tampere, Finland; 3 Department of Rehabilitation, Tampere University Hospital, Tampere, Finland; 4 Department of Radiology, Tampere University Hospital and University of Tampere, Tampere, Finland; 5 Tays Research Services, Wellbeing Services County of Pirkanmaa, Tampere University Hospital, Tampere, Finland

**Keywords:** Infundibular dilatation, Follow-up, Aneurysm, Subarachnoid hemorrhage

## Abstract

**Purpose:**

Although infundibular dilatations (IDs) have been thought to be benign anatomical variants, case reports suggest that they can grow and rupture. The aim of this study was to determine whether IDs have a tendency to grow or rupture.

**Methods:**

The study population was collected from the Tampere University Hospital (TAUH) Aneurysm Database. The presence of IDs was screened from the medical records and imaging studies of 356 intracranial aneurysm patients left to follow-up from 2005 to 2020. The imaging studies were reviewed to confirm the IDs, and their clinical course. Finally, we performed a systematic review of published cases of ID leading to aneurysmatic rupture from PubMed.

**Results:**

We found 97 typical IDs in 83 patients and 9 preaneurysmal lesions resembling ID in 9 patients. Out of the typical cone-shaped IDs, none grew or ruptured in a total follow-up of 409 patient-years. One preaneurysmal lesion ruptured during a follow-up: this lesion had components of both infundibular dilatation and aneurysm at the beginning of follow-up. In the systematic literature search, we found 20 cases of aneurysmatic SAHs originating from an ID. Of those, only 7 had imaging available prerupture. All 7 IDs were typically cone-shaped, but a branching vessel originating from the apex of ID was only seen in 4/7.

**Conclusion:**

Typical infundibular dilatations seem to be benign anatomical variants that are stable and, thus, do not need prophylactic treatment or imaging follow-up. Likely, the SAHs reported from IDs were actually caused by misdiagnosed preaneurysmal lesions.

**Supplementary Information:**

The online version contains supplementary material available at 10.1007/s00701-024-05890-w.

## Introduction

**Fig. 1:**
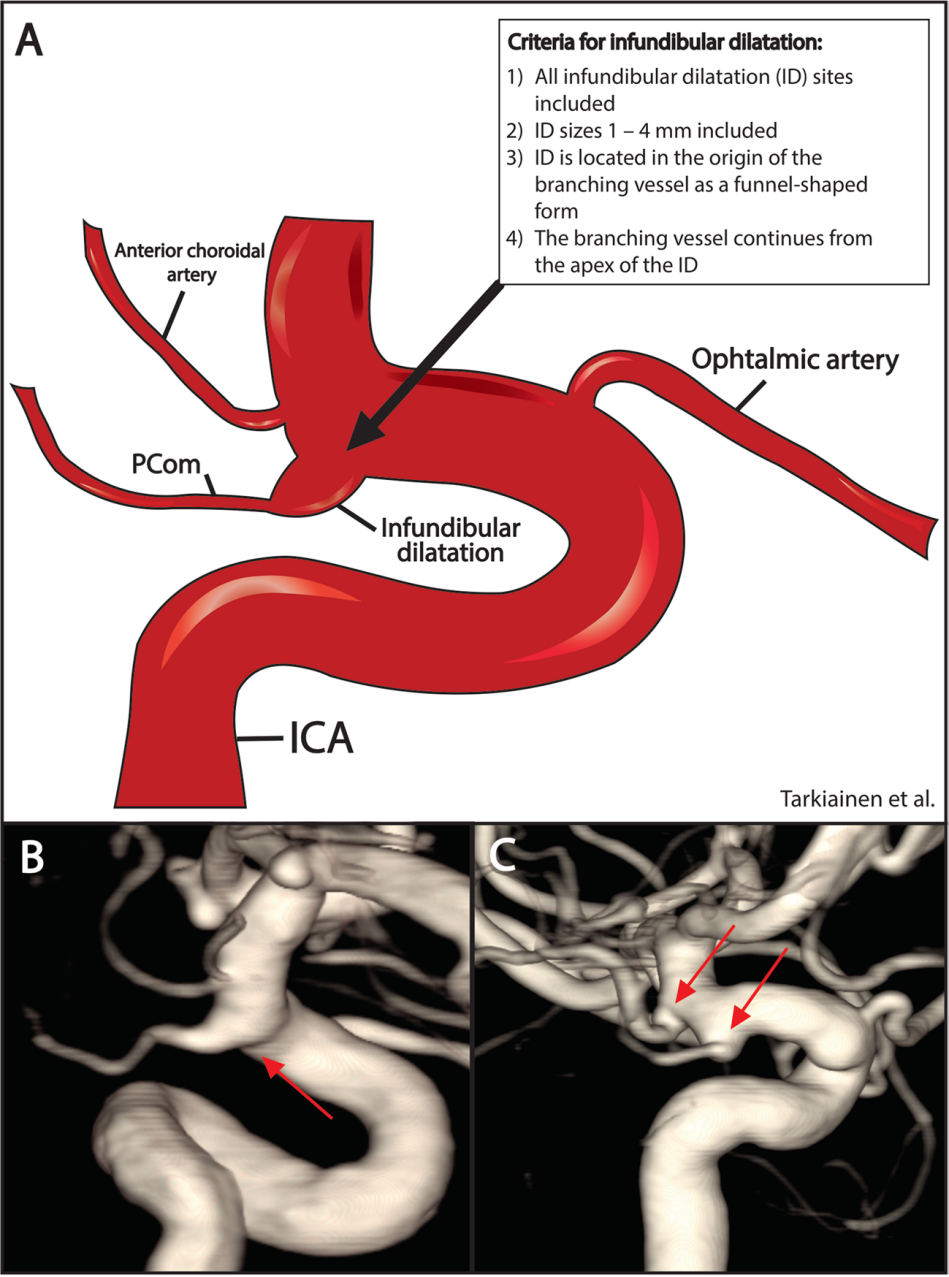
**A** Illustration of ID and the definition criteria we used to diagnose them and **B** example of PCom ID diagnosed at DSA and **C** example of multiple IDs diagnosed in DSA. The IDs are located on the PCom and on the anterior choroidal artery. The arrows in the figure point to the IDs

Infundibular dilatations (IDs) are funnel-shaped enlargements of the origin of a cerebral artery (Fig. 1). They are most often located in the ICA origin of posterior communicating artery (PCom), but they can also be present in other cerebral arteries. Other common locations are the arteries that originate from the ICA or MCA, such as anterior choroidal artery (AChoA) or perforating arteries of MCA. IDs can also be found in the small arteries of other anterior or posterior circulation.

**Fig. 2:**
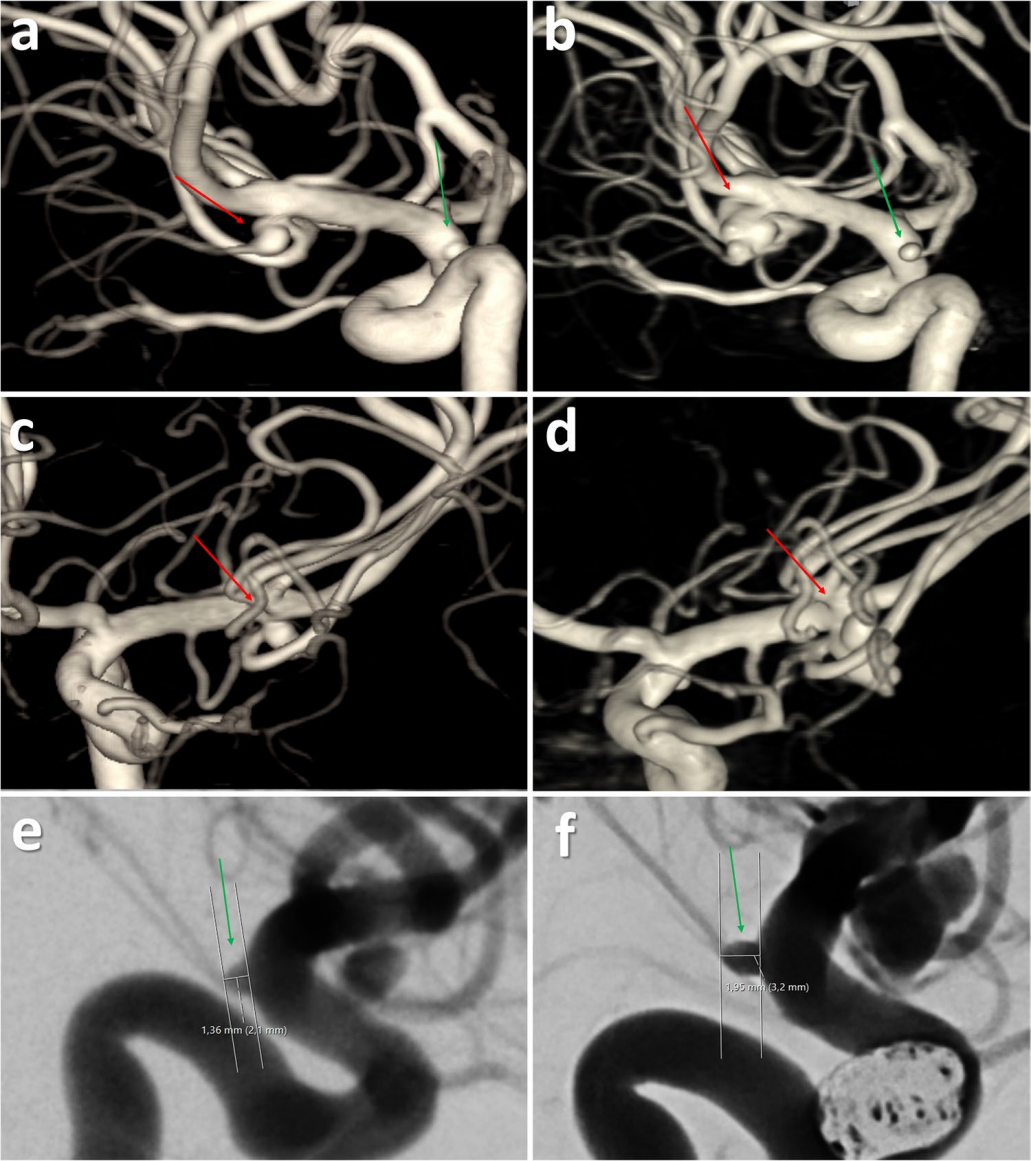
Example of a preaneurysmal lesion resembling ID that ruptured. The left column represents the DSA images taken in 2015 (**a**, **c**, **e**) and the right column represents the DSA images taken in 2023 at the time of the rupture (**b**, **d**, **f**). Contrary to the typical ID, this lesion in the MCA bifurcation had two branching arteries originating from the dilatation (**a**, **c**). The growth of the ruptured lesion can be seen in panels b and d (red arrow). In addition to the growth and rupture of the preaneurysmal lesion, the patient also had aneurysm growth on PCom aneurysm (**e**, **f**). The red arrows point to the lesion that ruptured, and the green arrow points to the aneurysm growth

Currently, IDs are thought to be benign anatomic variants [8]. However, some case reports suggest that IDs should be considered preaneurysmal lesions with a risk of developing into an aneurysm or even rupture on its own [6, 14]. Recently, at our institution (Tampere University Hospital), a preaneurysmal lesion with components of infundibular dilatation ruptured 8 years after its diagnosis (Fig. 2). This lesion was not a typical aneurysm either, as it had two branching vessels originating from the dome, resembling an ID.

Triggered by this patient case, we performed a systematic review of reported cases of subarachnoid hemorrhage originating from IDs. This investigation revealed similar cases, thereby prompting a deeper exploration into whether IDs should indeed be redefined as potential precursors of aneurysms instead of anatomical variants. The primary aim of this study is to firstly determine whether infundibular dilatations tend to grow in follow-up and secondly to determine whether these infundibular dilatations preceded the arterial bifurcations that subsequently developed de novo aneurysms.

## Material and methods

**Fig. 3:**
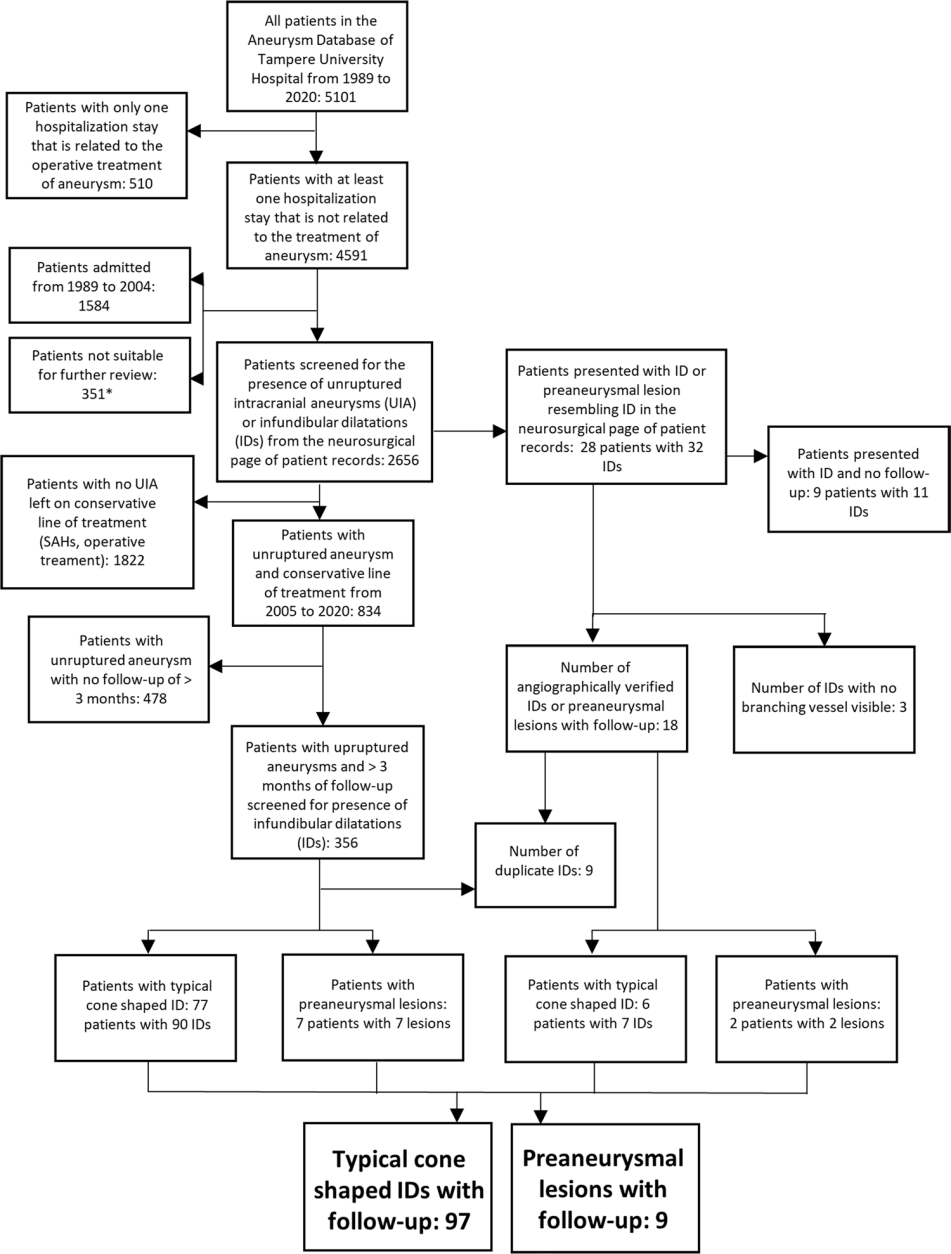
A flowchart describing how the study cohort was formed. *The group that was not suitable for further review (*n* = 351) included mostly patients who had had SAH and a conservative line of treatment or patients for whom medical records were not available

This dataset was collected along the collection of a cohort of unruptured aneurysms. All patients admitted between 2005 and 2020 with potential unruptured aneurysm were screened (i.e., at least one hospitalization not related to operative treatment of aneurysm). TAUH implemented the use of a digital image archive in 2003–2004. Since 2005, the imaging studies are available consistently and therefore that was chosen as the starting point for this study. The data for hospitalization information and baseline characteristics were collected from the TAUH Aneurysm Database which is a database into which all patients who presented with subarachnoid hemorrhage (ICD I60.1-9) or unruptured aneurysm (ICD I67.1) in its catchment area are entered. The imaging studies of patients followed for unruptured intracranial aneurysms were reviewed for the presence of IDs and the clinical course of these IDs during the imaging follow-up. In addition, the medical records of patients with more than one visit to TAUH related to an unruptured aneurysm were screened for any record of a diagnosed ID (Fig. 3).

### Definition of the infundibular dilatation

The ID diagnosis in our patient cohort was based on the report of an experienced radiologist, but in addition, the imaging studies of all patients with UIAs left to follow-up were reviewed, to confirm the presence of an ID. Currently, a proper definition of the ID has been established for the PCom location of the ID. In this study, the following criteria were used to identify the classical cone-shaped IDs.

1. All sites of ID included
2. ID sizes 1–4 mm included
3. ID is located in the origin of the branching vessel as a funnel-shaped form
4. The branching vessel continues from the apex of the ID

Moreover, we chose to include a separate analysis of preaneurysmal lesions that resembled IDs. In the patient’s medical records, these lesions were classified as preaneurysmal lesions or aneurysms. We chose to include these lesions since they had components of IDs, and they were similar to the cases of ruptured IDs presented in the literature. The abnormality was classified as preaneurysmal lesion if any of the following criteria were met.

1. More than one branching vessel originating from the ID
2. Branching vessel originating from the wall before the apex
3. Any irregularities in the shape

### Measurement of infundibular dilatation size and growth

The maximum size, neck, height, and diameter of the branching vessel from the ID were measured from the computed tomography angiography, digital subtraction angiography, or magnetic resonance angiography, as reported previously [22]. All images were available in digital form, and all measurements were made from the digital images. The size of the ID and branching vessel were measured on a scale of 0.1 mm. The primary outcome for this study was stability of the ID, meaning either (1) stable ID, (2) rupture, or (3) growth in any of the observed directions (e.g., height, width). Growth was defined as an increase in ID size by 1.0 mm. Follow-up time was defined as the time from diagnosis to the most recent available imaging study.

### Retrospective angiographic analysis of arteries that developed de novo aneurysms

De novo aneurysms were collected from the TAUH Aneurysm Database in two ways. Firstly, aneurysms classified as de novo were identified from the Aneurysm Database from the period 1989–2020. Secondly, to include all de novo aneurysms, patients with unruptured aneurysms were inspected, and if one patient had multiple aneurysms and different times of diagnosis for them, there would have been formation of de novo aneurysm and the latter aneurysm was included as de novo. The search from the TAUH Aneurysm Database yielded 41 de novo aneurysms, and the search from the unruptured aneurysms file yielded 19 aneurysms. After the removal of duplicates (*n* = 2) the imaging studies of patients with a total of 58 de novo aneurysms were retrospectively reviewed to confirm true de novo aneurysm formation and to confirm the morphology of arteries in which the de novo aneurysms formed before de novo aneurysm formation. A total of 21 de novo aneurysms diagnosed onwards 2005 were included in the final analysis of arteries that developed de novo aneurysms. The patients with no prior imaging studies available before the de novo aneurysm formation were not included as the presence of ID could not be reviewed.

### Collection of clinical variables

All the clinical variables such as age, history of aneurysms, and risk factors were collected retrospectively from the patient’s medical records. In TAUH, the clinical information is collected during every hospitalization by the attending physician.

### Systematic review of the literature

The PubMed database was searched with the terms (“infundibular dilation” OR “infundibulum”) AND (“hemorrhage” OR “rupture” OR “bleeding”). In addition, the reference lists of prior publications on ruptured infundibular dilatations were systematically screened (Supplementary Figure 1). The search from the PubMed and from the reference lists was conducted between 1 August 2023 and 3 August 2023.

### Eligibility criteria, selection process, and data extraction

A study was eligible for our analysis if the following criteria were fulfilled: (1) the study addresses ruptured IDs and (2) the article was in the English language. Studies describing ID evolution to aneurysm, and studies not retrievable were excluded. All records were screened, and abstracts and full texts were assessed by one author (JT). The review was reported according to the preferred reporting items for systematic reviews and meta-analyses (PRISMA) 2020 checklist [18].

Data were extracted and recorded in an Excel spreadsheet (Version 2307, Microsoft) by one author (JT). The data extracted included the study title, author names, publication year, patient demographics, and morphological features of the IDs. In addition, the published radiological image representing preruptured IDs and ruptured IDs were retrieved from the publications as available.

The risk of bias was assessed with Joanna Briggs Institute (JBI) Critical Appraisal Checklist for Case Reports or with JBI checklist for case series [9] by one author (JT).

### Statistical analysis

The statistics were calculated using SPSS version 26.0 statistical software (IBM). Proportions and percentages were calculated for categorical variables. The means, medians and ranges were calculated for continuous variables. The results of systematic review are presented in tabular format. Proportions and percentages were calculated for the characteristics of ruptured IDs presented in the literature. Subgroup analysis was performed on those IDs with images available before rupture.

## Results

### Characteristics of the study cohort

Table 1 presents the baseline characteristics of the study cohort. During our study period, 83 patients with 97 typical IDs and 9 patients with 9 preaneurysmal lesions resembling infundibulum were identified from the TAUH Aneurysm Database. Out of the cohort of patients with UIAs left for follow-up, 90 IDs were found in 77 patients (Fig. 3), suggesting that the prevalence of IDs is 21.6% (77/356) in patients with aneurysms.

**Table 1:** Characteristics of the study cohort and infundibular dilatations*.* *The number of patients followed for the stability of aneurysm is smaller as all patients did not have unruptured aneurysms (Fig. 3)

	Patients with typical cone-shaped IDs (*n* = 83)	Patients with preaneurysmal lesions (*n*= 9)
Age, median (IQR)	56 years (49–65 years)	58 years (47–68 years)
Gender (% women)	62.7% (52/83)	66.7% (6/9)
Aneurysm	98.8% (82/83)	88.9% (8/9)
Multiple aneurysms	65.1% (54/83)	66.7% (6/9)
Aneurysm growth during follow-up	6.5% (5/77) *	0.0% (0/7) *
Aneurysm rupture during follow-up	3.9% (3/77) *	0.0% (0/7) *
Prior SAH	27.7% (23/83)	22.2% (2/9)
Smoking history	67.5% (56/83)	44.4% (4/9)
Hypertension	59.0% (49/83)	55.6% (5/9)
Family history of aneurysms	13.3% (11/83)	22.2% (2/9)
	**Typical cone-shaped IDs (*n*** **= 97)**	**Preaneurysmal lesions (*n*= 9)**
Length of follow-up (median, IQR)	3.0 years (1.5–6.2 years)	4.6 years (1.5–8.2 years)
Follow-up over 5 years	34.0% (33/97)	44.4% (4/9)
Follow-up over 7 years	20.6% (20/97)	11.1% (1/9)
Total follow-up	409.3 years	49.6 years
Growth during follow-up (%)	0.0% (0/97)	11.1% (1/9)
Rupture during follow-up (%)	0.0% (0/97)	11.1% (1/9)
ID characteristics		
Neck width (mean)	2.6 mm	2.8 mm
Height (mean)	1.9 mm	2.8 mm
Largest size (mean)	2.6 mm	3.1 mm
Branching vessel diameter (mean)	0.7 mm	1.0 mm
Neck width/branching vessel diameter ratio, median (range)	3.7 (2.1–10.0)	2.9 (2.3–4.3)
Parent artery		
PCom	79.4% (77/97)	22.2% (2/9)
MCA	4.1% (4/97)	55.6% (5/9)
ACom	2.1% (2/97)	-
PCA	2.1% (2/97)	-
ACA	2.1% (2/97)	-
AChoA	6.2% (6/97)	-
Ophthalmic artery	2.1% (2/97)	22.2% (2/9)
Other small ICA perforants	2.1% (2/97)	-

*SAH*, Subarachnoid hemorrhage; *PCom*, posterior communicating artery; *MCA*, middle cerebral artery; *ACom*, anterior communicating artery; *PCA*, posterior cerebral artery; *PICA*, posterior inferior cerebellar artery; *ACA*, anterior cerebral artery; *AChoA*, anterior choroidal artery

The median age for patients with typical ID was 56 years and 62.7% (52 of 83) were women (Table 1). Almost all (98.8%, 82 of 83) of these patients also had an aneurysm in addition to the ID. Two patients had no aneurysms: one in the typical ID group and one in the preaneurysmal lesion group. They were included in the Aneurysm Database, as they were initially thought to be aneurysms before diagnosis in subsequent more precise imaging studies. 67.5% (56 of 83) of patients in the typical ID group had a history of smoking (current or past), and 59.0% (49 of 83) had hypertension. Out of the patients with typical IDs, 77 patients were concomitantly followed for the stability of UIAs with the same methods. In contrast to the stable IDs in 77 patients, 6.5% (5 of 77) of the patients had aneurysm growth, and 3.9% (3/77) had aneurysm rupture in the same follow-up period of 409.3 patient-years.

### Characteristics and stability of infundibular dilatations

By far the most common location for typical IDs was the origin of the PCom (79.4%, 77 of 97), followed by the AChoA IDs (6.2%, 6 of 97) and MCA IDs (4.1%, 4 of 97) (Table 1). The mean neck size was 2.6 mm, the mean height was 1.9 mm, and the mean largest size was 2.6 mm. The mean branching vessel diameter was 0.7 mm, and the median neck width to branching vessel diameter ratio was 3.7.

None of the typical IDs showed any signs of growth or rupture in a total follow-up of 409.3 patient-years. 34.0% (33 of 97) of patients had a follow-up of over 5 years. Total follow-up for preaneurysmal lesions was 49.6 patient-years, and one of them ruptured after 8 years of follow-up. The diagnostic modalities used to identify and follow the IDs are presented in Supplementary Table 1.

### Lack of infundibular dilatations in bifurcations that eventually developed saccular intracranial aneurysms

To confirm that typical IDs are not the precursor state of aneurysm formation, we reviewed the imaging studies of the cerebral vasculature of patients who later developed 21 de novo aneurysms. The median age of patients who presented with de novo aneurysms was 47 years (range 22–61), and the median follow-up to de novo aneurysm formation was 5.3 years (IQR 4.5–6.5 years). Eleven of 21 (52%) of confirmed de novo aneurysms formed at a site of bifurcation and 10 of 21 (48%) formed at the sidewall of the parent artery. In the bifurcations in which aneurysms developed, no IDs were seen in the bifurcation site prior to de novo aneurysm formation. No IDs were neither seen at the site of de novo aneurysm formation of sidewall aneurysms.

### Systematic review of published cases of ID rupture

The systematic search yielded 104 studies from PubMed and from the reference lists (Supplementary Figure 1). Seventy studies were excluded after reviewing title/ abstracts. After assessing the eligibility from full texts of 34 studies, 21 were excluded. Full text was not available for 7 studies, and 14 studies did not match the inclusion criteria. Eight studies were not suitable, three studies assessed the growth of IDs [6, 15, 26], one article was in the Japanese language [21], one was a hemodynamic study of stable IDs [1], and one study was a systematic review [8]. Finally, a total of 20 cases of ruptured IDs were included from 13 individual studies (Table 2, Supplementary Figures 2-5). Twelve studies were case reports, and one study was a case series. All the ruptured IDs were located at the PCom. The median age for ID rupture was 53 years, and 70% (14/20) were female. Images of the ID before rupture were available for 7 of the subsequently ruptured IDs. Of those 7 IDs, all were typical cone-shaped lesions at diagnosis. However, the branching vessel that should originate from the apex, could be visualized in only 4/7 of the IDs. Out of the IDs that ruptured in a follow-up, all of them had had previous SAHs from other aneurysms (5/7) or from unknown origin (2/7). Furthermore, de novo aneurysm formation was seen in 4 out of 7 patients in the time interval between ID diagnosis and rupture.

**Table 2:** Previously reported ruptures of infundibular dilatations

Author and publication year	Sex	Age	Prerupture images available	Rupture images available	Initially typical ID shape	Typical ID shape at rupture	Branching vessel visible	De novo aneurysm formation	Prior SAH
Laurent 2020 [13]	Female	55	No	Yes	-	No	No	-	No
Lee 2021 [14]	Male	27	No	Yes	-	Yes	Yes	-	No
	Female	46	No	Yes	-	Yes	Yes	-	No
	Female	76	Yes	Yes	Yes	No	Yes	Yes	Yes
	Female	70	No	No	-	-	-	-	-
	Female	83	No	No	-	-	-	-	-
	Female	78	No	No	-	-	-	-	-
	Female	78	No	No		-	-	-	-
Zolnourian 2019 [28]	Female	59	No	Yes	-	Yes	No	-	No
Karakezi 2014 [11]	Female	60	Yes	Yes	Yes	No	Yes	Yes	Yes
Cowan 2014 [4]	Male	23	Yes	Yes	Yes	Yes	No	No	Yes
Yu 2010 [27]	Male	35	No	Yes	-	Yes	Yes	-	No
Coupe 2006 [3]	Male	51	No	Yes	-	Yes	Yes	-	No
Radulovic 2006 [19]	Female	34	Yes	Yes	Yes	No	No	No	Yes
	Female	38	Yes	Yes	Yes	No	No	No	Yes
Kuwahara 2001 [12]	Female	67	No	Yes	-	Yes	Yes	-	No
Ohyama 1994 [17]	Male	57	No	Yes	-	Yes	No	-	No
Itakura 1983 [10]	Female	33	Yes	Yes	Yes	No	Yes	Yes	Yes
Trasi 1981 [24]	Female	26	No	Yes	-	Yes	Yes	-	No
Stuntz 1970 [20]	Male	38	Yes	Yes	Yes	No	Yes	Yes	Yes

## Discussion

In this study, we report the first large-scale follow-up study of 97 typical infundibular dilatations and 9 preaneurysmal lesions in patients with aneurysms. All the typical IDs remained stable in a total follow-up of 409.3 patient-years. One preaneurysmal lesion ruptured after 8 years of follow-up.

### The ruptured lesion resembling both infundibular dilatation and aneurysm

In our series, the one ruptured lesion resembling an aneurysm and ID (Fig. 2) remained the only one showing instability. This ruptured lesion was not a typical infundibular dilatation: conventional IDs are symmetrical, cone-shaped dilations at the origin of the vessel. In contrast, this ruptured lesion initially resembled an aneurysm, except for the branching vessels originating from it. This lesion was thought to be the source of the SAH since the bleeding pattern was typical for a ruptured MCA bifurcation lesion. Moreover, the location and clinical presentation differed from a perimesencephalic SAH [23]. Besides the ruptured lesion, the patient also presented aneurysm instability during follow-up. Similarly, the literature review showed that out of the 7 IDs that ruptured during a follow-up, 4 patients also had de novo aneurysm formation. These are interestingly high rates of de novo aneurysm formation since it is estimated that only 2% of patients with ruptured or unruptured aneurysm develop de novo aneurysms in a mean follow-up of 8.3 years [7]. This may indicate that the patients presenting with ruptured IDs may have some unknown factors in the cerebral vasculature that predispose to an aneurysm formation/instability in an unusual manner.

Based on the systematic review of the SAHs originating from the IDs, we found that many of the ruptured IDs reported in the literature, would not have been classified as IDs based on the criteria, we used to assess whether a lesion is ID or aneurysm. In patients with available imaging studies, only 6/16 would have been classified as IDs at the time of rupture. Of those patients with imaging available prerupture, 4 out of the 7 would have been classified as IDs due to the lack of branching vessel originating from the apex of ID in 3/7 of IDs. This raises the question of whether the ruptured IDs presented in the literature were IDs or preaneurysmal lesions or aneurysms.

### Nature of classical infundibular dilations as anatomical variants—not as precursors of intracranial aneurysms

Considering the reported cases of SAHs caused by the rupture of IDs as shown in Table 2, there is a growing concern regarding whether the traditional interpretation of IDs as benign anatomical variants should be revised. Since a limited number of follow-up studies of diagnosed IDs have been published, this topic merits to be studied, especially since even the largest published follow-up study (32 IDs in children followed for 86 patient-years) by Dmytriw et al. [5] is alone underpowered to make firm conclusions (Data supplement). In comparison to the results of Dmytriw et al., we found that a great majority of IDs were located in the PCom origin, as reported previously in the literature [8]. This difference in the proportion of PCom IDs may be attributed to unintentional selection bias. Their study cohort consisted of children, who were overrepresented with conditions that predispose to the development of vascular abnormalities, as acknowledged by Dmytriw et al. [5].

Given the high (3%) prevalence of IAs in the past middle-aged population [25], it seems expected that IAs would occasionally develop at sites of IDs even without any causal relationship. This can well explain the published cases in which IA formation occurred at sites of ID (Table 2). To demonstrate that bifurcations with IDs develop fewer IAs than is expected overall, the number of studied cases needs to be over 150 in order to reach a statistical power of 80% with an alpha level of 0.05, if one expects that 3% of cerebral artery bifurcations would develop IAs and only 0.1% of IDs would develop IAs (Data supplement). Of course, the 3% prevalence of IAs does not signify that in the patient with IAs all cerebral artery bifurcations would have IAs, so in fact the number of cases needed to obtain sufficient statistical power is much more than 150. Hence, our cohort adds more certainty to the results published by Dmytriw et al., though even combined the two studies are somewhat underpowered.

Our patient series combined with that of Dmytriw et al. show with a reasonable degree of certainty that typical cone-shaped IDs do not have an increased risk of progressing into aneurysms. This supports the traditional concept that IDs are anatomical variants that develop in the early stage of vascular development and that the processes for ID formation seem to be different from the pathophysiological processes underlying aneurysm formation. One explanation of the origin of IDs could be that they are remnants of fetal PCA that originates from the ICA at around 28 days in the embryological development [16]. In addition, if IDs develop in the early stages of vascular development, it is surprising that in patients who later developed an aneurysm, the aneurysm formed somewhere else and not in the ID, as observed in our series. If IDs form during early vascular development and they are prone to developing aneurysms, one would expect that aneurysms would specifically develop in these locations.

In our study cohort, most of the followed patients with typical ID (98.8%, 82/83) had also an aneurysm, and 65.1% (54/83) had multiple aneurysms. This indicates that our study population was more susceptible to developing vascular malformations than the general population, as these patients had already developed an aneurysm. Moreover, UIA instability (growth or rupture) was seen in 10.4% (8/77) of patients with IDs, which are typical rates of UIA instability [2]. As none of the typical IDs grew or ruptured, their clinical course seems to be different from aneurysms. Our second observation that cerebral artery bifurcations which eventually developed sIAs (de novo aneurysms), were initially anatomically “normal” without any IDs, provides further support for this interpretation.

### Should infundibular dilations routinely undergo radiological follow-up?

In our institution, routine follow-up of IDs has not been the practice. However, when the patient has in another location a true sIA that requires radiological follow-up, as a byproduct the concomitant IDs are followed—as in our study cohort. Since our results imply that typical IDs do not progress, the rational indication for radiological follow-up of IDs would be only to confirm that the followed lesion truly is an ID in an unclear situation. In such a case, however, a higher resolution imaging could give the diagnostic clarity faster, and avoid the subsequent psychological load imposed on the patient by the uncertainty of having a potentially growing aneurysmatic lesion. It seems that only typical cone-shaped IDs should be considered as IDs. When the dilation does not have a vessel arising from the tip of the fundus, or when it does not fulfill other criteria of a typical ID or has aneurysm-like features, more detailed imaging studies or image follow-up are warranted. Since our study includes only 9 preaneurysmal lesions resembling ID, out of which only one turned unstable, the precise growth rate of these lesions remains uncertain. We suggest that the first follow-up study of preaneurysmal lesion should be performed 1 year after the initial imaging study. If the lesion has remained stable, another imaging study should be performed two years after the previous one.

Since IDs seem to be anatomical variants that arise during the genesis of vasculature, we do not think that IDs in children and adults should behave differently. Thus, we do not recommend follow-up for typical cone-shaped IDs in children. However, if it is uncertain whether a radiological change is ID or preaneurysmal lesion, we recommend a similar follow-up as described for adults.

### Strengths and limitations

To the best of our knowledge, this is the first large-scale follow-up study of infundibular dilatations in the adult population. Furthermore, this study specifically concentrates on patients affected by aneurysms. This emphasis has particular significance due to emerging concerns about whether IDs could potentially be precursors of aneurysms and should they be treated.

This study had some limitations. Firstly, the imaging follow-up was short for some of the IDs. We chose to include all IDs with follow-up of over 3 months, as the knowledge of the clinical course of IDs is very scarce. However, even though the imaging follow-up is short for some IDs, none of them ruptured and therefore were not referred to TAUH at the time of the data collection (8/2023). Despite the short follow-up time in some patients, we had several patients followed for longer periods (Table 1).

Secondly, the imaging follow-up was carried out with multiple imaging modalities (Table S1), which may reduce the accuracy of the size measurements. However, if there had been a significant change in ID size, one would have been able to notice it despite the different modalities. To address this limitation, we chose to measure IDs with two modalities if they were performed in a time span of one week in 9 IDs. The mean difference in size was 0.2 mm, which is rather low. It seems that the larger bias comes from the measurement itself. A growth of 1 mm is an uncertain finding anyway, since by measuring any 3D object from different 2D directions, the results are easily off by 1 mm. Because of this, IDs were measured from as similar projections as possible when measuring the size from follow-up studies and from the initial imaging studies. We conclude that the comparison of different imaging modalities during follow-up did not impair the detection of possible growth or change in shape any more than what would have been a reasonable detection limit when comparing similar imaging modalities at different time points.

Thirdly, the study design (Fig. 3) excludes the potential IDs in patients with only one hospitalization related to operative treatment of the aneurysm. However, as these patients had only one hospitalization stay, there would not have been any imaging follow-up for these patients and, thus, were not suitable for a follow-up study.

Fourthly, the systematic review part of the study was conducted by a single author. The single-author approach may introduce potential bias, primarily because it limits the inclusion of diverse perspectives. In addition, the review and the protocol were not registered.

## Conclusions

Classical funnel-shaped infundibular dilatations seem to be benign anatomical variants that do not have tendency to grow or rupture. Patients presented with typical IDs do not require prophylactic treatment or imaging follow-up.

## Supplementary information

ESM 1 (DOCX 5.22 MB)

## Data Availability

Following the GDPR regulations of the European Union, the data used in this study cannot be made freely available. Pseudonymized dataset can, however, be shared after formal approval of a scientific research plan and data management plan by the local Ethical review board and Tampere University Hospital. All data used in the systematic review are provided in the Table 2 and in the Data supplement.
