# Supplementary material for: Stability of infundibular dilatations: a single center follow-up study and systematic review of the literature
Source: Acta Neurochir (Wien). 2024 Jan 30;166(1):48. doi: 10.1007/s00701-024-05890-w (PMC10824818; doi:10.1007/s00701-024-05890-w)
Supplement: Supplementary file 1 — (DOCX 5.22 MB) [file 701_2024_5890_MOESM1_ESM.docx]

Data supplement

**Stability of infundibular dilatations. A single center follow-up study and systematic review of the literature**

Jeremias Tarkiainen BM^1,2^, Liisa Pyysalo MD PhD ^2,3^, Tero Hinkka MD ^4^, Juha-Pekka Pienimäki ^4^, Antti Ronkainen MD PhD ^1^ and Juhana Frösen MD PhD^1,2.5^

1) Dept of Neurosurgery, Tampere University Hospital and University of Tampere, Finland

2) Hemorrhagic Brain Pathology Research Group, Faculty of Medical Technology and Health Sciences, Tampere University, Tampere, Finland

3) Dept of Rehabilitation, Tampere University Hospital, Tampere, Finland

4) Dept of Radiology, Tampere University Hospital and University of Tampere, Tampere, Finland

5) Tays Research Services, Wellbeing Services County of Pirkanmaa, Tampere University Hospital, Tampere, Finland

Table S1. The diagnostic modalities used to identify and follow infundibular dilatations.

|  | **Typical cone shaped IDs (n = 97)** | **Preaneurysmal lesions (n= 9)** |
| --- | --- | --- |
| **Modality at diagnosis** |  | |
| DSA | 40.2 % (39/97) | 22.2 % (2/9) |
| CTA | 42.3 % (41/97) | 44.4 % (4/9) |
| MRA | 17.5 % (17/97) | 33.3 % (3/9) |
| **Modality at first follow-up** |  | |
| DSA | 35.1 % (34/97) | 44.4 % (4/9) |
| CTA | 21.6 % (21/97) | 22.2 % (2/9) |
| MRA | 43.3 % (42/97) | 33.3 % (3/9) |
| First follow-up | 100.0 % (97/97) | 100.0 % (9/9) |
| **Modality at second follow-up** |  |  |
| DSA | 14.4 % (14/97) | - |
| CTA | 15.5 % (15/97) | 22.2 % (2/9) |
| MRA | 36.1 % (35/97) | 33.3 % (3/9) |
| Second follow-up | 66.0 % (64/97) | 55.6 % (5/9) |
| **Modality at third follow-up** |  | |
| DSA | 7.2 % (7/97) | 11.1 % (1/9) |
| CTA | 12.4 % (12/97) | - |
| MRA | 14.4 % (14/97) | 22.2 % (2/9) |
| Third follow-up | 34.0 % (33/97) | 33.3 % (3/9) |
| **Modality at fourth follow-up** |  | |
| DSA | 2.1 % (2/97) | - |
| CTA | 7.2 % (7/97) | - |
| MRA | 2.1 % (2/97) | 11.1 % (1/9) |
| Fourth follow-up | 11.3 % (11/97) | 11.1 % (1/9) |
| **At least one DSA** | 68.0 % (66/97) | 55.6 % (5/9) |

*Quality assessment of studies included in the systematic review*

Among 12 case reports (Supplementary Table 2), 7 studies received a perfect score (8/8). Six of the studies had a score of 6/8. The reporting of patient’s demographics and medical history received the lowest scores. The one case series study (Supplementary Table 3) received a perfect score in the quality assessment (10/10).

Table S2. Quality assessment of the case report studies.

| First author name, publication year | Q1 | Q2 | Q3 | Q4 | Q5 | Q6 | Q7 | Q8 | Total |
| --- | --- | --- | --- | --- | --- | --- | --- | --- | --- |
| **Laurent 2020** | No | No | Yes | Yes | Yes | Yes | Yes | Yes | 6 |
| **Zolnourian 2019** | No | No | Yes | Yes | Yes | Yes | Yes | Yes | 6 |
| **Karakezi 2014** | Yes | Yes | Yes | Yes | Yes | Yes | Yes | Yes | 8 |
| **Cowan 2014** | Yes | Yes | Yes | Yes | Yes | Yes | Yes | Yes | 8 |
| **Yu 2010** | No | No | Yes | Yes | Yes | Yes | Yes | Yes | 6 |
| **Coupe 2006** | Yes | Yes | Yes | Yes | Yes | Yes | Yes | Yes | 8 |
| **Radulovic 2006 case 1** | Yes | Yes | Yes | Yes | Yes | Yes | Yes | Yes | 8 |
| **Radulovic 2006 case 2** | Yes | Yes | Yes | Yes | Yes | Yes | Yes | Yes | 8 |
| **Kuwahara 2001** | Yes | Yes | Yes | Yes | Yes | Yes | Yes | Yes | 8 |
| **Ohyama 1994** | No | No | Yes | Yes | Yes | Yes | Yes | Yes | 6 |
| **Itakura 1983** | No | No | Yes | Yes | Yes | Yes | Yes | Yes | 6 |
| **Trasi 1981** | Yes | Yes | Yes | Yes | Yes | Yes | Yes | Yes | 8 |
| **Stuntz 1970** | No | No | Yes | Yes | Yes | Yes | Yes | Yes | 6 |

Q1. Were patient’s demographic characteristics clearly described?

Q2. Was the patient’s history clearly described and presented as a timeline?

Q3. Was the current clinical condition of the patient on presentation clearly described?

Q4. Were diagnostic tests or assessment methods and the results clearly described?

Q5. Was the intervention(s) or treatment procedure(s) clearly described?

Q6. Was the post-intervention clinical condition clearly described?

Q7. Were adverse events (harms) or unanticipated events identified and described?

Q8. Does the case report provide takeaway lessons?

Table S3. Quality assessment of the case series studies.

| First author name | Q1 | Q2 | Q3 | Q4 | Q5 | Q6 | Q7 | Q8 | Q9 | Q10 | Total |
| --- | --- | --- | --- | --- | --- | --- | --- | --- | --- | --- | --- |
| **Lee 2021** | Yes | Yes | Yes | Yes | Yes | Yes | Yes | Yes | Yes | Yes | 10 |

Q1. Were there clear criteria for inclusion in the case series?

Q2. Was the condition measured in a standard, reliable way for all participants included in the case series?

Q3. Were valid methods used for identification of the condition for all participants included in the case series?

Q4. Did the case series have consecutive inclusion of participants?

Q5. Did the case series have complete inclusion of participants?

Q6. Was there clear reporting of the demographics of the participants in the study?

Q7. Was there clear reporting of clinical information of the participants?

Q8. Were the outcomes or follow up results of cases clearly reported?

Q9. Was there clear reporting of the presenting site(s)/clinic(s) demographic information?

Q10. Was statistical analysis appropriate?


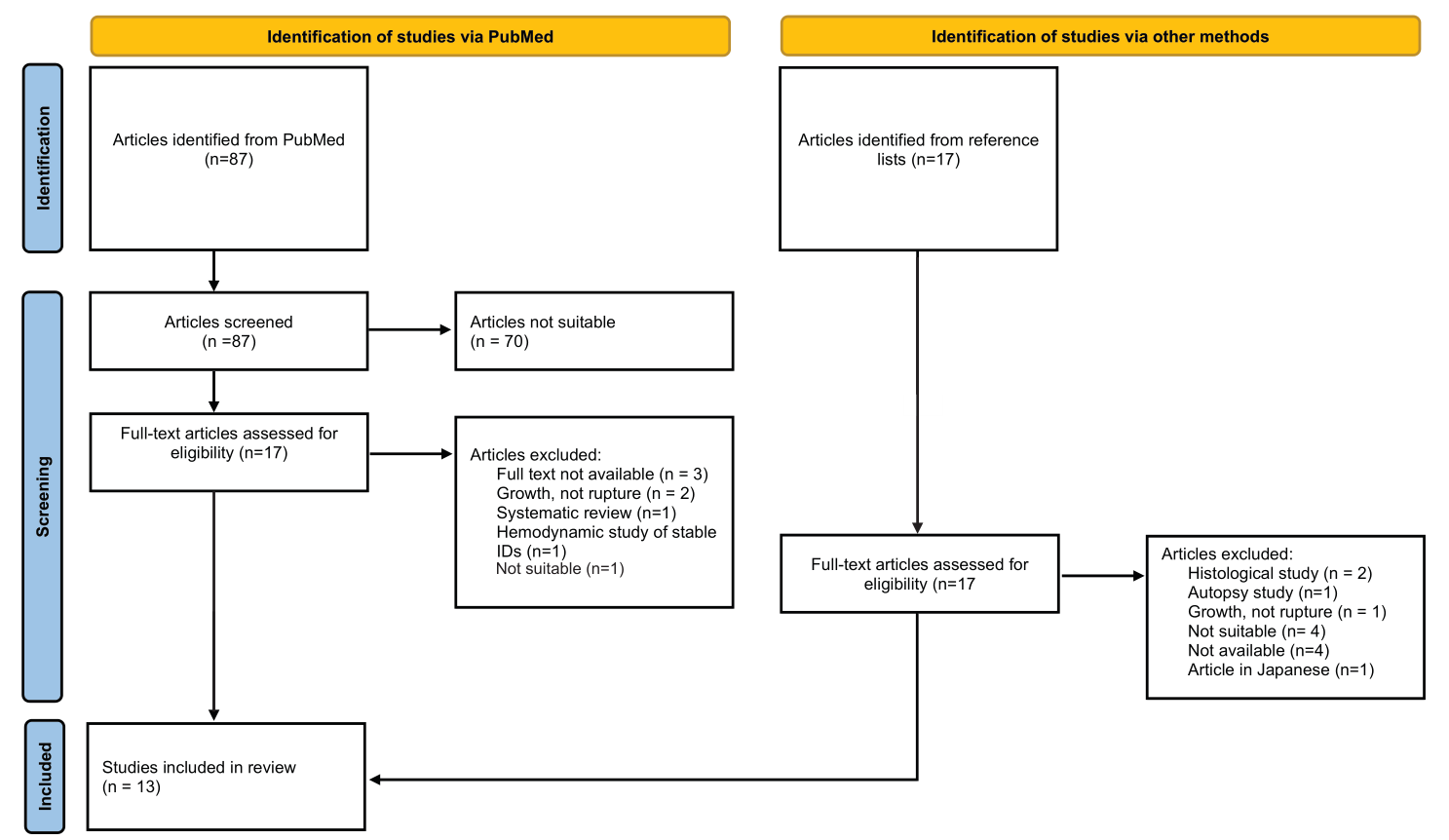


Figure S1. PRISMA flowchart of studies included in the systematic review.


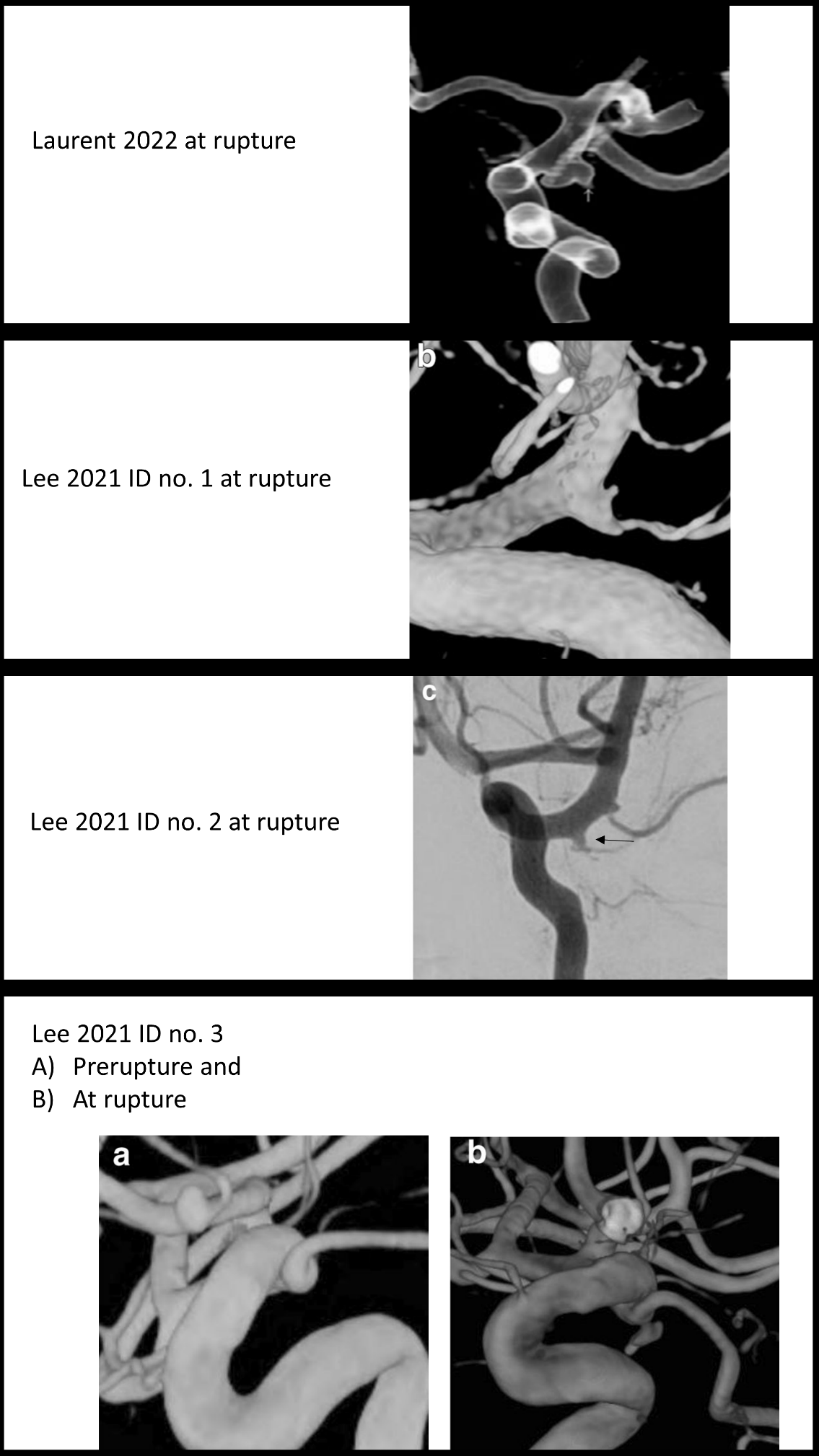


Figure S2. Ruptured IDs no. 1-4 presented in the literature.


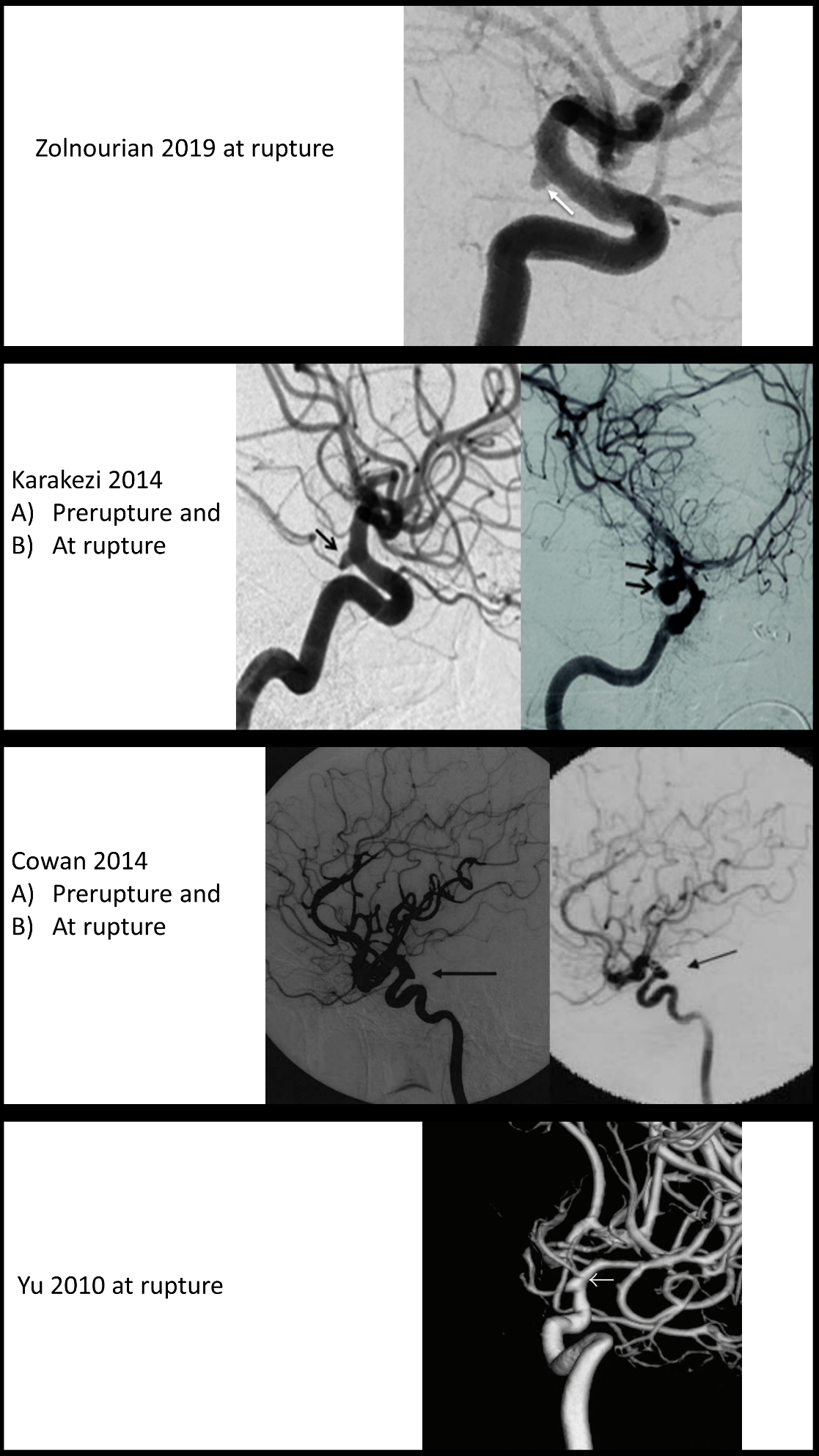


Figure S3. Ruptured IDs no. 5-8 presented in the literature.


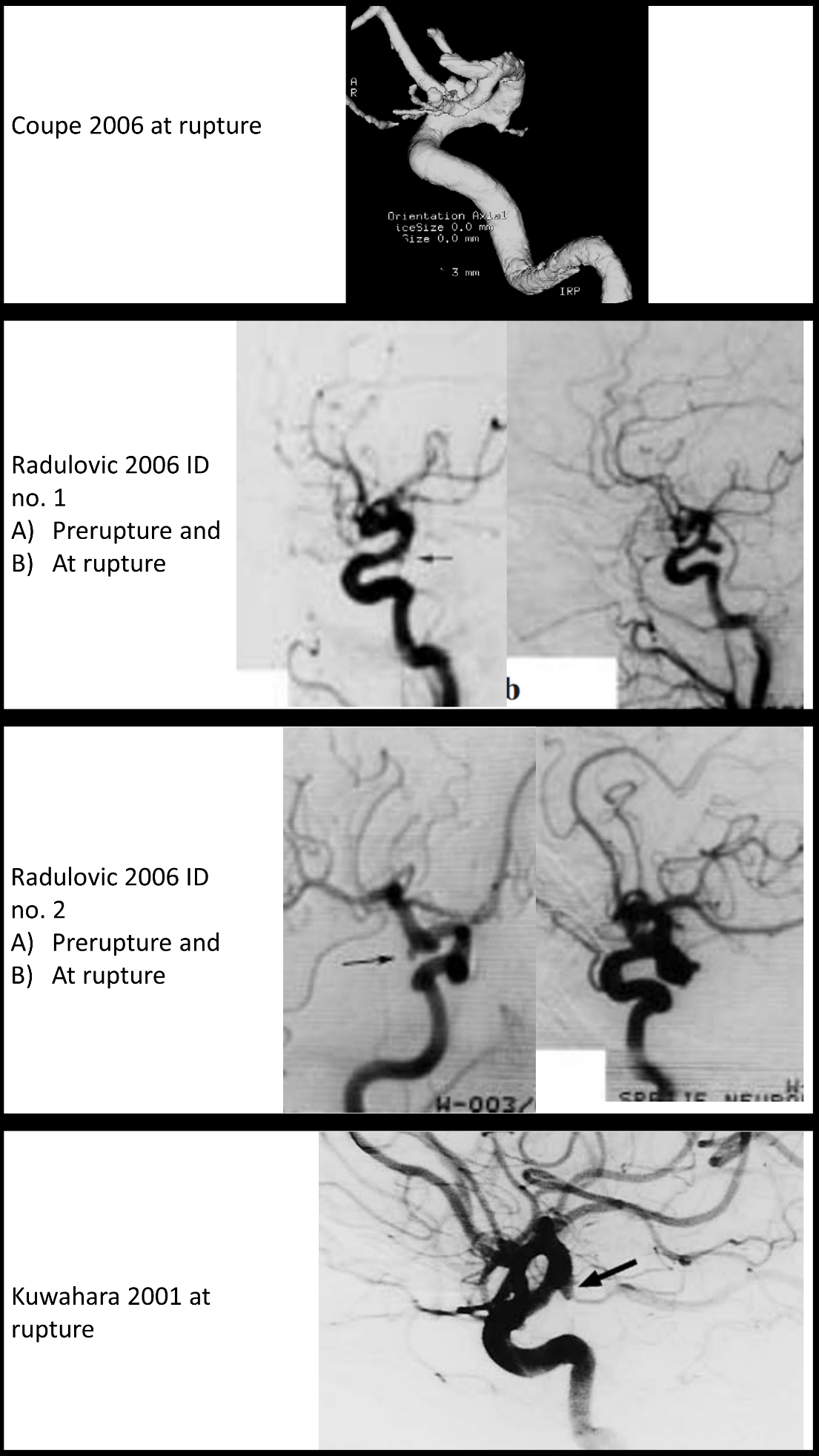


Figure S4. Ruptured IDs no. 9-12 presented in the literature.


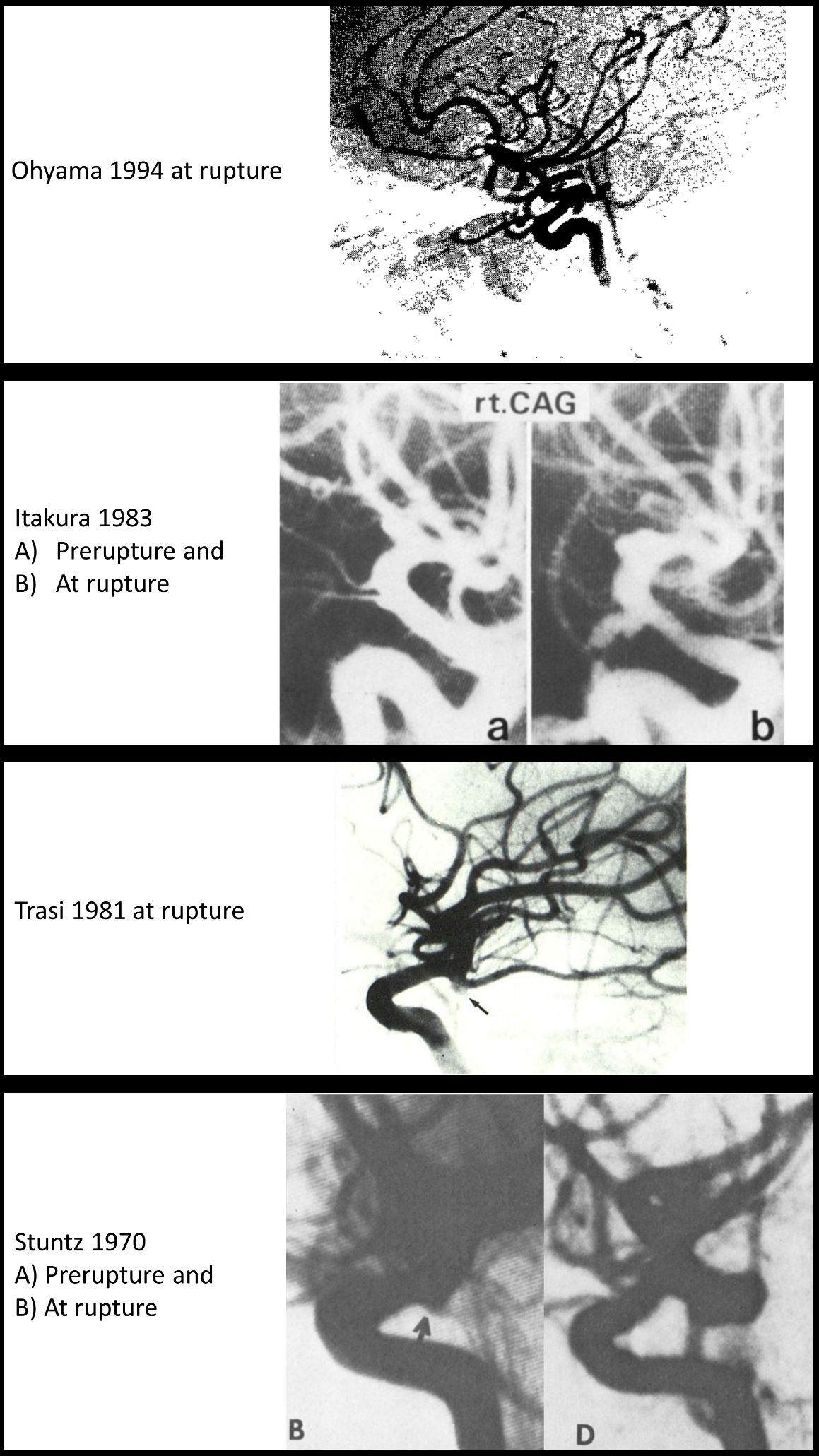


Figure S5. Ruptured IDs no. 13-16 presented in the literature.


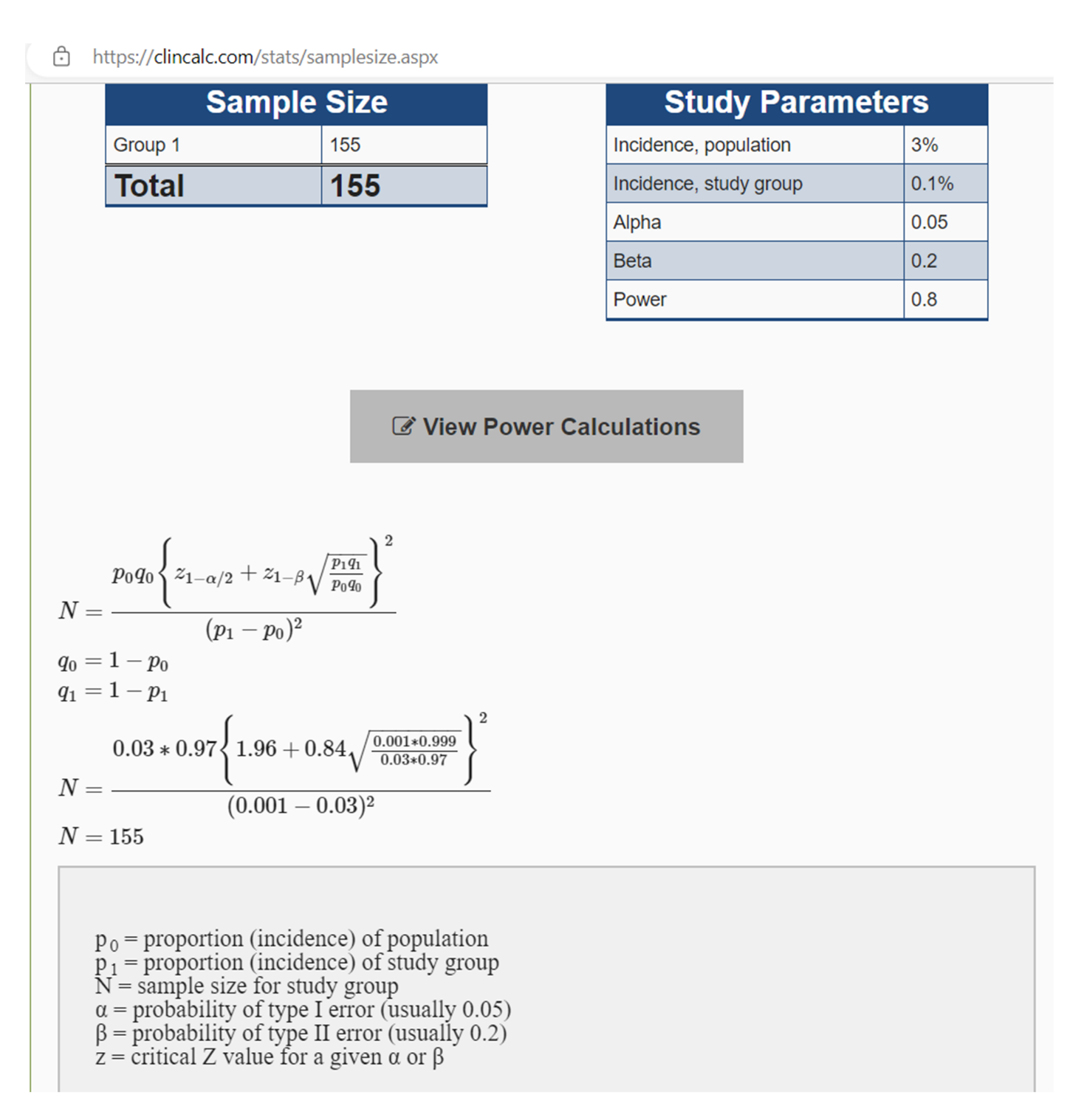


Figure S6 summarizes the power calculation made with the assumptions that i) the general incidence of intracranial aneurysms would be 3% of the population, ii) that intracranial aneurysms would develop in less than 0.1% of infundibular dilatations, and that iii) the alpha level (likelihood of type I error) would be 0.05 and iv) statistical power 0.8 (1-likelihood of type II error).
